# The Philadelphia Practice of Midwifery

**Published:** 1840

**Authors:** 


Art. XIX.—*The Philadelphia Practice of Midwifery.* By Charles D. Meigs, M. D., Philadelphia, 1838. 1 vol. 8vo. pp. 370.

The author of this unpretending little volume has been extensively and favorably known to the profession in this country since 1831, by his translation of Velpeau's Midwifery. He has been engaged for several years in the practice and teaching of obstetrics, and the work before us, we are informed in the dedication, is rather an expression of his own reflections and experience than a dry compilation of materials to be found every where in the obstetric library, and as such he hopes it may be found serviceable to *students.* We do not doubt that his hope will be realized, and we venture to add that *practitioners*, who have enjoyed less favorable opportunities than the author for keeping up with the progress of the science, and acquiring a practical acquaintance with its important subjects, will derive from its perusal more precise views than they have hitherto possessed, and much useful information. Not that we are able to say that the author has exhibited much, if any originality, (for who, indeed, can lay claim to such distinction at this day, in a science which, as Velpeau expresses it in the introduction to the second edition of his "*Traite Complet de l' art des Accouchemens*," derives its essential principles from the laws of mechanics or is founded upon exact anatomy, giving to it a precision approaching the certainty of the mathematical sciences,) but he has shown himself familiarly acquainted with all that has been discovered or written on the subjects which he discusses. The easy, unaffected, yet chaste and correct style that is observed throughout the book, together with its freedom from learned parade, and the perfect candour and good faith that appear conspicuous on every page, impart to it a charm that is quite captivating, and force the conviction that we are communing with an accomplished teacher and a most estimable man. Such a work as the present, if we do not greatly err, was much needed in this country; the writings of Dr. Dewees, which occupy a tutelary rank in the library of almost every practitioner, excellent as they are in many respects, are liable to some very weighty objections. Among these, not the least is the space allotted to critical disquisitions of little practical moment, and a general diffusiveness, which, when employed upon trival matters, becomes irksome to the reader. This is a fault that does not attach to Dr. Meigs' book; indeed, it may be questioned if certain important subjects are not despatched with too great brevity, as, for example, puerperal convulsions. In the main, however, we think the author has succeeded in furnishing a compendium of obstetrics, which will be highly prized by students, in attendance on a course of lectures, and by practitioners, as a book of reference. Again, the system of Dr. Dewees, (but this is no fault of his,) contains some errors of doctrine and practice, which have been disclosed since he wrote. In embryology he adopts substantially the views of the Hunters, which have been implicitly received by almost all writers and teachers until a very recent period. It is but a short time, for example, since the correctness of John Hunter's account of the structure of the placenta was called in question, and yet, if any faith may be given to the most careful inquiries of Velpeau, Lee, and others, it was founded upon a delusion resulting from the manner in which the great physiologist attempted to investigate it. Certainly, if the placenta be examined in situ, and without doing violence to its structure by forcing injections of wax into it, nothing like a *maternal* and *fœtal* portion will be discovered, nor will large arteries be seen passing from the uterus into pretended cells in its substance. The placenta belongs, in truth, exclusively to the fœtal system, and is composed entirely of its bloodvessels, arteries and veins, connected by cellular tissue, and has no vascular connexion with the uterus, save by capillary vessels, and the existence of even these is doubted or denied by Velpeau. It may be regarded, then, as the capillary termination of the vascular system of the fœtus, in apposition with the uterus for the purpose of deriving from thence its supplies of nutriment and oxygen, or whatever is imparted in respiration.

The observations of Dr. Meigs go to confirm the truth of this doctrine ; he has had more than one opportunity of witnessing the detachment of the placenta, by peeling off the uterus, first laid open by an incision from it fundus to near the os uteri, and upon the most careful examination, with the eyes directed to the line where the separation was taking place, *not a single blood-vessel was discovered passing from the placenta to the womb, or from the womb to the placenta. The slightest force only was required to effect the detachment, and the adhesion was not near so firm as is often observed betwixt the skin and a good adhesive plaster.*

If it be asked of what utility is a knowledge of the true structure of the placenta ; we answer that at least one curious phenomenon is erroneously explained, and one practical recommendation predicated upon the commonly received doctrine of Hunter. One of the sounds, *bruit de soufflet*, detected by obstetric auscultation, has been commonly supposed to be owing to the utero-placental circulation—the passage of the blood through large vessels between the uterus and placenta. This explanation, however, cannot be admitted for the bruit de soufflet has been known to persist after the death of the fœtus from procidence of the umbilical cord, and as long as forty-four hours after delivery. Moreover, we are assured that it has been discovered when pregnancy did not even exist, the vascular system of the uterus being in a state of morbid development and activity. The practical suggestion, to which we alluded, is that of Professor Hamilton, of Edinburg, who advises astringent injections, per vaginam, in flooding from separation of the placenta implanted over the os uteri. According to him, the ruptured utero-placental arteries are speedily retracted within the substance of the uterus, under favor of their peculiar spiral extremities, and soon cease to pour out blood ; but the circulation still going on between the uterus and the attached portion of the placenta, blood escapes from the detached portion, on account of the free communication between its imaginary cells. To restrain such a hemorrhage, one of his indications is, by styptics topically applied, to constringe or alter the texture of the separated portion of the placenta, that it may not allow the blood to escape. Such a practice, founded upon mistaken notions of the structure and economy of the placenta, it is to be apprehended is worse than useless; by removing coagula it will favor the continuance of hemorrhage.

The majority of his readers, we are sure, will feel obliged to Dr. Meigs for his attempts to appreciate the true value of a much used and abused agent in obstetrical practice—we mean the *tampon.* As a means of arresting uterine hemorrhage, occurring during pregnancy and parturition, it is frequently resorted to, because the discharge may seem to be too profuse to be trusted to medicinal treatment and to require something to mechanically obstruct its flow, and allow the blood to coagulate in the mouths of the bleeding vessels. Whoever employs the tampon, with such a vague apprehension of its powers and of the indications for its use, will often be disappointed in the effects which he expected to derive from it; for, the effusion of blood may be going on internally when its escape is effectually prevented, or the tampon, while it controls the hemorrhage, may provoke the premature expulsion of the fœtus. In all cases of *accidental* hemorrhage in the latter months of gestation, therefore, we concur with Dr. Meigs in the proscription of the tampon. We can have no security against fatal internal bleeding in such cases, because the retention of the blood necessarily increases the extent of the separation between the membranes and uterus, and this involves an aggravation of the hemorrhage. But in

*unavoidable* hemorrhage, we do not, with Dr. M., apprehend any such risk ; nether reasoning nor observation, as far as we have been able to collect its testimony, justifies the belief that the placental attachment over the os uteri is in danger of being disrupted by the damming of the blood in the vagina, so as to allow its insinuation between the membranes and uterus, even to its fundus. In these alarming, though fortunately rare cases, we have a valuable auxiliary in the tampon ; with it we can control the discharge, without fear of internal hemorrhage, until the condition of the os uteri will justify manual delivery. There is another reason why the tampon should be employed in *unavoidable*, and withheld in *accidental* hemorrhage, derived from one of its properties which Dr. Meigs has taken particular pains to describe, viz: its power of inducing or invigorating expulsive contractions of the uterus. The implantation of the placenta over the os uteri, as a general rule, precludes the hope of the pregnancy being preserved until term ; as gestation advances and the expansion of the cervix progresses, the patient is liable, at longer or shorter intervals, to attacks of flooding, which will at last become so profuse as to leave no other alternative than delivery. Much of the safety of this measure will depend upon the manner in which it is conducted ; *it should never be made an exclusively artificial process.* To forcibly dilate the os uteri, while it is rigid or but little inclined to yield, and to rudely pass the hand into the cavity of the uterus and extract the fœtus, while it evinces no disposition to co-operate by parturient contractions, would greatly magnify the danger, and cruelly aggravate the sufferings of the patient. The most judicious writers, insist, therefore, with great earnestness, on the propriety of refraining from delivery until the os uteri is dilated or easily dilatable, i. e. until the parturient process is in fact instituted. Now, if the tampon, by its stimulation of the os uteri, does excite uterine contraction, of which we have no doubt, ought it not always to be used in these cases with this view, if there were no other, that the natural parturient agents may be awakened, and art be allowed only to follow and assist, instead of leading and performing the chief labor? Such an excitant of parturient action is the more to be desired in these critical cases, because it is well known that it is not spontaneously established commonly, until the patient has been brought into great peril from repeated losses of blood, and, if left alone, it is probable that she would sink from hemorrhage before the tardy powers of nature would come to her rescue. Hence, practitioners, who reject the tampon as a present means of restraining the effusion of blood, find themselves often obliged to deliver before the os uteri is sufficiently dilated or dilatable, to render the operation easy or safe, and, if much violence is done in gaining admission for the hand, the patient is no better off than if she had been left to nature.

Entertaining the views which have been thus succinctly expressed, with regard to the modus operandi of the tampon, we give our ready assent to the correctness of the doctrine that, "it is not a remedy for those cases in which any hope is yet entertained of saving the pregnancy;" but we contend for its applicability as well as utility in placenta prævia cases, because no such hope can be entertained. With this single exception we think Dr. Meigs' observations on the tampon as valuable as they will probable be novel to some of his readers. Certainly, no one who will dispassionately examine the subject, will think of using the tampon on account of hemorrhage in threatened abortion, as long as there is just ground of hope that the catastrophe can be averted; but if abortion be inevitable it might become useful to employ it, even though the hemorrhage be not alarming, to facilitate the completion of the process.

It was not possible for the author, within the limits of so small a volume, to treat of all the diseases peculiar to females, or, perhaps, to do greater justice than he has to such as he has selected. His observations on some of the derangements of menstruation are good as far as they go; we regret that it was not compatible with his plan to dwell at greater length on the different forms of amenorrhea and to include, also, menorrhagia, even at the sacrifice of his disquisition on generation, a subject more speculative than practical, concerning which so much has been written and so little is known. Dr. Meigs remonstrates very forcibly against the injustice that has been done to derangements of the menstrual functions in the empirical treatment which they have received from physicians, as if they did not come within the purview of enlightened and rational pathology. Although Dr. Dewees has done much to correct this fault in American practitioners, it is to be feared that too many, who are careful to probe other diseases to the bottom, are still content to prescribe for amenorrhea, dysmenorrhea, and menorrhagia, *by name* or after the most superficial examination.

It is just as essential to successful practice in these affections to investigate the pathological state of the generative organs, and to attend to the state of the other organs, which can influence them by sympathy, as in any other class of diseases which the physician is called to combat. No fact in medicine is better established than that amenorrhea, for example, may be connected with precisely opposite morbid conditions of the uterus and general system; requiring in the one case antiphlogistic, and in the other tonic treatment. We do not, however, subscribe to the doctrine of the constitutional origin of uterine affections, to the extent that Dr. Meigs seems inclined to go. This doctrine, which has the sanction of very high authority in this country as well as in Europe, may be stated in the language of Dr. Meigs: "It is not to be supposed that if a woman's constitution can be brought into healthful play in all other regards, she will be vicious or disordered in this instance of amenorrhea. I grant that sudden arrests or stoppage, may take place from slight and perhaps local causes; but I speak now of the instances of rebellious obstructions. I wish to impress the idea that a woman is not unhealthy because she fails to menstruate, but rather, that she fails to menstruate because she is unhealthy." Now, what evidence is there that the uterine organs enjoy such an immunity from the direct disturbance of morbid causes. We should be greatly amazed were it asserted that, if healthful vigour be imparted to a woman's constitution, disease would be eradicated from any other organ that might happen to be diseased in its functions. Let us suppose, for example, that the lungs are the seat of disease, and that there is morbid or suppressed secretion of the bronchial mucous membrane—will remedies addressed alone to the constitution always reach or overcome the disease? We opine not. What proof have we that the uterus is more amenable to constitutional sanative influences than the lungs? But the supposition is that the uterus is not really diseased—that it only complains on account of general bad health, and that being remedied, it resumes its function as a matter of course. Now, while we admit that a woman may cease to menstruate because she is unhealthy, we stoutly maintain that far more frequently she loses her health in consequence of ceasing to menstruate. We do not mean that there is any wonderful health-preserving efficacy in the periodical sanguineous evacuation, or that the immediate or remote constitutional derangements attendant on amenorrhea are produced by the suppression in itself considered; but the torpor, irritation, or inflammation of the uterus, which is the real disease, undermines the general health through the medium of the numerous and important sympathies, by which it is so closely connected with the other viscera of the system.

The subject of labor, which should, of course, be the burden of a treatise on the practice of midwifery, is very fully and satisfactorily discussed by Dr. Meigs. His account of it as a physiological act is generally accurate, and his directions for its conduct are such as the most enlightened experience must sanction. The student cannot be too earnestly exhorted to make himself thoroughly conversant with the physiology of labor, and especially its mechanism, as by such knowledge alone can he be prepared to comprehend or surmount the obstacles that more frequently embarrass it, than ignorant or indolent practitioners are willing to allow. When labor is retarded, how often do we see such accoucheurs console themselves for the total darkness in which they are enveloped by the comfortable prophecy that nature will at last prevail, when by rendering, it may by the slight assistance that is needed, the patient might have been saved hours of present suffering and the future risk, which such protraction involves. We have already expressed the opinion that such a reinforcement as Dr. Meigs' book has furnished to the standard authority in this country, was wanted, because it corrects certain errors of doctrine and practice. In further justification of this opinion, we may here allude to the management of face presentations. According to Dr. Dewees and most of the English writers, these presentations are to be regarded as preternatural, and treated by bringing down the vertex, by the crotchet or forceps. Now, although manual delivery should not be shrunk from, when absolutely required, it is always to be considered as an operation that is painful and not free from danger, especially in the hands of an inexperienced or unskilful practitioner, and as such it should be avoided whenever it is possible, consistently with the safety of the patient. Attempts to push up the face to have it replaced by the vertex, as the records of writers who have pursued such practice show, have greatly bruised and disfigured the features, stripped off the skin, gouged the eyes, &c., to such a degree, that the substitution of the crotchet would have been merciful. The recital of such shocking barbarities makes us shudder, even though we might be convinced of the necessity of their commission ; but when we come to learn by painful experience that they were unnecessary—that face presentations fall within the pale of natural labor and are but little less favorable, for mother and child, then vertex positions, while we rejoice in the knowledge, we grieve to mark the bloody tracks of our art by which it was so slowly acquired.

Dr. Meigs is clearly of opinion that face cases may well be included among the natural labors, except some failure in the powers of the woman should cause us to convert them into preternatural ones, by obliging us to turn and deliver by the feet; to restore the vertex by some serious operation; or to extract with the forceps. For the establishment of this among the maxims of obstetrics, science and humanity are chiefly indebted to Madame Lachapelle, the midwife-in-chief of the Paris Maternite ; it has since been received and defended by the greatest number of the French writers, who have adduced in its defence an amouut of evidence that is wholly irresistible. According to the observations of many of these, the child was expelled by the unaided powers of nature in a larger proportion of face presentations than was witnessed by Madame Lachapelle; in the practice of some of them the assistance of art was not found necessary in a single case.

Such, also, was the experience of Dr. Collins in the Dublin Lying-in Hospital, wherein thirty-three cases of face presentations occurred during his mastership; four of the children were still-born, *all were delivered without assistance.*

With the chapter on "presentation of the pelvic extremity of the fœtus," we are much pleased, with the exception of an item of practice, peculiar to the author, we believe, and of questionable propriety, to say the least. It is well known that the most critical time in these labors, as regards the safety of the child, is when the head comes to be engaged in the pelvis. Should any considerable delay arise in this stage of its expulsion, the child will be in imminent danger of perishing from asphyxia. To guard against this, it is Dr. Meigs' unfailing custom to order his forceps to be placed within reach in every case of breech or footling labors, in order to be enabled to extract the head without delay, and he is persuaded that the consequence of this care has been the saving of several lives that must have been lost but for this precaution. This practice, we think, cannot be commended to general imitation; a resort to instruments is surely unnecessary in a majority of these cases, and it is difficult, in a moment of anxiety and alarm, to resist the temptation to employ them if they be at hand. We should rather say to young accoucheurs at least, let your instruments be far from you in managing breech presentations, lest you be tempted to potter with them to the detriment of the mother without benefit to the child. Besides, we are persuaded that with a little adroitness, the requisite degree of force can be applied on the superior maxilliary bone and base of the occiput, to extract the head without the slightest risk of injury to the child; and until this can be deliberately accomplished, asphyxia may be prevented by pressing back the perineum to admit air to the child's mouth, in the manner recommended by Dr. Meigs. By this expedient the child may be enabled to breath and cry for a longer period, (Dr. Meigs says for 20 or 25 minutes,) than the head will be detained in the pelvis once in fifty cases, if the previous management have been judicious; i. e. if the expulsion of the body be committed to the natural powers, and the head be suffered to come down into the pelvis with the proper degree of flexion, and with the face towards the hollow of the sacrum.

But we must bring this rambling notice to a close, having already extended it to greater length than we intended; not that we grudge Dr. Meigs' book the space we have allowed it—we honestly think it deserving of more—but it is within the digits and purses of all our readers, and doubt not they will examine it for themselves. H. M.

